# Complete Genome Assembly of *Staphylococcus epidermidis* AmMS 205

**DOI:** 10.1128/genomeA.01059-14

**Published:** 2014-11-06

**Authors:** K. W. Davenport, H. E. Daligault, T. D. Minogue, K. A. Bishop-Lilly, S. M. Broomall, D. C. Bruce, P. S. Chain, S. R. Coyne, K. G. Frey, H. S. Gibbons, J. Jaissle, C. L. Redden, C. N. Rosenzweig, M. B. Scholz, H. Teshima, S. L. Johnson

**Affiliations:** a Los Alamos National Laboratory (LANL), Los Alamos, New Mexico, USA; b Diagnostic Systems Division (DSD), United States Army Medical Research Institute of Infectious Diseases (USAMRIID), Fort Detrick, Maryland, USA; c Naval Medical Research Center (NMRC)-Frederick, Fort Detrick, Maryland, USA; d Henry M. Jackson Foundation, Bethesda, Maryland, USA; e United States Army Edgewood Chemical Biological Center (ECBC), Aberdeen Proving Ground, Maryland, USA

## Abstract

*Staphylococcus epidermidis* causes a large number of catheter-related sepsis infections annually in the United States. We present the 2.54-Mbp complete genome assembly of reference strain *S. epidermidis* AmMS 205, including a single 37.7-kbp plasmid. The annotated assembly is available in GenBank under accession numbers CP009046 and CP009047.

## GENOME ANNOUNCEMENT

*Staphylococcus epidermidis* has been called an “accidental pathogen,” because it evolved to be a commensal rather than pathogenic microbe (1). Unlike *Staphylococcus aureus*, *S. epidermidis* produces only a small number of toxins and exoenzymes that cause tissue damage and its ability to act as an opportunistic pathogen depends on its ability to form biofilms that escape immune response (2). Despite the opportunistic nature of *S. epidermidis* infection, treatment costs in the United States alone are estimated at $2 billion annually (1). In this effort, we sequenced and assembled the genome of reference strain *S. epidermidis* AmMS 205 (ATCC 49134; CDC 73-57501).

High-quality genomic DNA was extracted from a purified isolate using Qiagen Genome Tip-500 at USAMRIID-DSD. Specifically, a 100-mL bacterial culture was grown to the stationary phase and nucleic acid was extracted as per the manufacturer’s recommendations. Sequence data included both 100-bp Illumina standard data (307-fold genome coverage) and long-insert 454 paired-end data (8,237 ± 2,059-bp insert, 29-fold genome coverage) (3, 4). The two datasets were assembled together in Newbler (Roche), and the consensus sequences were computationally shredded into 2-kbp overlapping fake reads (shreds). The raw reads were also assembled in Velvet, and those consensus sequences were computationally shredded into 1.5-kbp overlapping shreds (5). Draft data from all platforms were then assembled together with Allpaths, and the consensus sequences were computationally shredded into 10-kbp overlapping shreds (6). We then integrated the Newbler consensus shreds, Velvet consensus shreds, Allpaths consensus shreds, and a subset of the long-insert read-pairs using parallel Phrap (High Performance Software, LLC). Possible misassemblies were corrected and some gap closure was accomplished with manual editing in Consed (7–9).

The complete genome assembly of *S. epidermidis* AmMS 205 includes a 2,500,626-bp circular chromosome (32.1% G+C content) and a 37,688-bp circular plasmid (27.3% G+C content) and was annotated using an Ergatis-based workflow (10) at LANL with minor manual curation. A total of 2,347 coding sequences (57 located on the plasmid) and 19 rRNA and 59 tRNA sequences were noted. At least 7 of the virulence factors noted by Otto (1) were found upon preliminary review of the annotation, all of which are located on the chromosome.

### Nucleotide sequence accession numbers.

The chromosome (CP009046) and plasmid (CP009047) are publicly available in the NCBI database.
